# The Association Between Serum Vitamin D and Lipid Levels in Patients With Depression: A Cross-Sectional Study

**DOI:** 10.31083/AP46265

**Published:** 2026-04-09

**Authors:** Lihua Yao, Honggang Lv, Zhaowen Nie, Wei Wang, Simeng Ma, Zhili Niu, Ying Wang, Lijun Kang, Dan Xiang, Wei Yuan, Hexiang Chen, Zhongchun Liu

**Affiliations:** ^1^Department of Psychiatry, Renmin Hospital of Wuhan University, 430060 Wuhan, Hubei, China; ^2^Department of Clinical Laboratory, Institute of Translational Medicine, Renmin Hospital of Wuhan University, 430060 Wuhan, Hubei, China; ^3^Department of Psychiatry, Yidu People's Hospital, 443000 Yichang, Hubei, China; ^4^Department of Anesthesiology, Renmin Hospital of Wuhan University, 430060 Wuhan, Hubei, China; ^5^Taikang Center for Life and Medical Sciences, Wuhan University, 430072 Wuhan, Hubei, China

**Keywords:** depression, vitamin D, lipids, association, clinical study

## Abstract

**Background:**

Vitamin D deficiency is prevalent among individuals with depression; however, clinical findings regarding this association have been inconsistent. Additionally, a significant proportion of depressed patients present with dyslipidemia, yet the interplay between vitamin D status, lipid metabolism, and depression remains poorly understood. We aimed to explore the role of vitamin D in depression and to investigate the potential associations between vitamin D status, lipid metabolism, and depressive symptoms.

**Methods:**

We recruited 412 first-episode, drug-naïve patients with depression and 180 age-matched healthy controls. Fasting venous blood samples were collected in the morning to quantify serum vitamin D and lipid profiles. Depressive symptoms were assessed on the day of blood collection using both the Patient Health Questionnaire-9 (PHQ-9) and the 17-item Hamilton Depression Rating Scale (HAMD-17). Spearman's rank correlation was employed to examine associations between serum vitamin D concentrations and depressive symptom severity. Binary logistic regression analysis was subsequently performed to identify potential risk factors for depression.

**Results:**

Compared with healthy controls, depressed patients had significantly lower serum vitamin D and high-density lipoprotein cholesterol (HDL-C) levels. This sex-specific pattern showed that male patients had lower vitamin D, while female patients had lower HDL-C. Spearman's correlation analysis revealed significant inverse correlations of vitamin D and triglyceride (TG) with PHQ-9 and HAMD-17 scores among depressed patients. Logistic regression analysis indicated that individuals with higher vitamin D levels had a reduced likelihood of depression compared with those with low vitamin D levels (adjusted odds ratio (OR) = 0.950, 95% confidence interval (CI): 0.920–0.982, *p* = 0.002). Similarly, subjects with elevated HDL-C levels were associated with a lower likelihood of depression relative to those with diminished HDL-C levels (adjusted OR = 0.317, 95% CI: 0.173–0.583, *p* < 0.001).

**Conclusion:**

Serum vitamin D and HDL-C levels were lower in patients with depression than in healthy individuals. Both vitamin D and HDL-C may be inversely associated with depression.

## Main Points

1. Vitamin D and high-density lipoprotein cholesterol (HDL-C) levels were lower in patients with depression than in healthy controls.

2. Vitamin D levels were negatively correlated with the severity of depressive symptoms.

3. Vitamin D and HDL-C may be inversely associated with depression.

## 1. Introduction

Serum vitamin D exerts a broad range of effects by binding to the vitamin D receptor, which is expressed in nearly all tissues and cells, including brain regions implicated in neuropsychiatric disorders [1, 2]. Several studies have reported an association between vitamin D deficiency and psychiatric or mood disorders [3, 4, 5, 6]. However, findings from randomized controlled trials (RCTs) on the efficacy of vitamin D supplementation in treating depression remain inconsistent [7, 8, 9, 10]. This discrepancy may arise from overlooked factors, such as vitamin D dosage, individual age and sex, recurrent depressive episodes, the influence of antidepressant medications, and comorbid conditions affecting vitamin D absorption and metabolism. Consequently, depression in relation to circulating vitamin D levels requires further validation.

Vitamin D is a fat-soluble vitamin whose active form, 1,25-dihydroxyvitamin D [1,25(OH)2D], regulates cellular differentiation and biosynthetic pathways, including lipid biosynthesis, via mitochondrial vitamin D receptors [11, 12]. Observational studies suggest that vitamin D deficiency is associated with unfavourable blood lipid profiles, with inverse correlations observed between vitamin D levels and total cholesterol (TC), low-density lipoprotein cholesterol (LDL-C), and triglyceride (TG) [13]. Lipids are highly abundant in neural tissues and play a crucial role in neurodevelopment [14]. Research has established connections between lipids and depression [15, 16, 17]. Notably, a Mendelian randomization study investigating lipids and depression indicated a potential causal relationship between triglycerides and depressive symptoms [18]. Lipid homeostasis contributes significantly to multiple interconnected processes governing mood regulation and suicidal behaviours, including serotonin neurotransmission [19, 20], neurogenesis [21], and neuroprotection against both excitotoxicity and systemic inflammation [22]. Consequently, disturbances in lipid metabolism are increasingly recognised as potential biomarkers for depression [23].

While the individual associations of vitamin D and lipid profiles with depression have been widely studied, their relative strength, comparative importance, and potential co-occurrence patterns within a homogeneous cohort of first-episode, drug-naïve patients remain unclear. Systematic evaluation of both biomarkers in this well-defined population helps to delineate a more comprehensive physiological profile of depression. In this study, we recruited a cohort of relatively young, drug-naïve, first-episode patients with depression without major physical comorbidities, along with carefully matched healthy controls. This design enables a clearer investigation of the relationship between vitamin D, lipids, and depression. Our work aims to clarify their association with the disorder and explore their potential role in its pathogenesis, thereby contributing novel insights for clinical strategy development.

## 2. Materials and Methods

### 2.1 Study Population

**Fig. 1.**
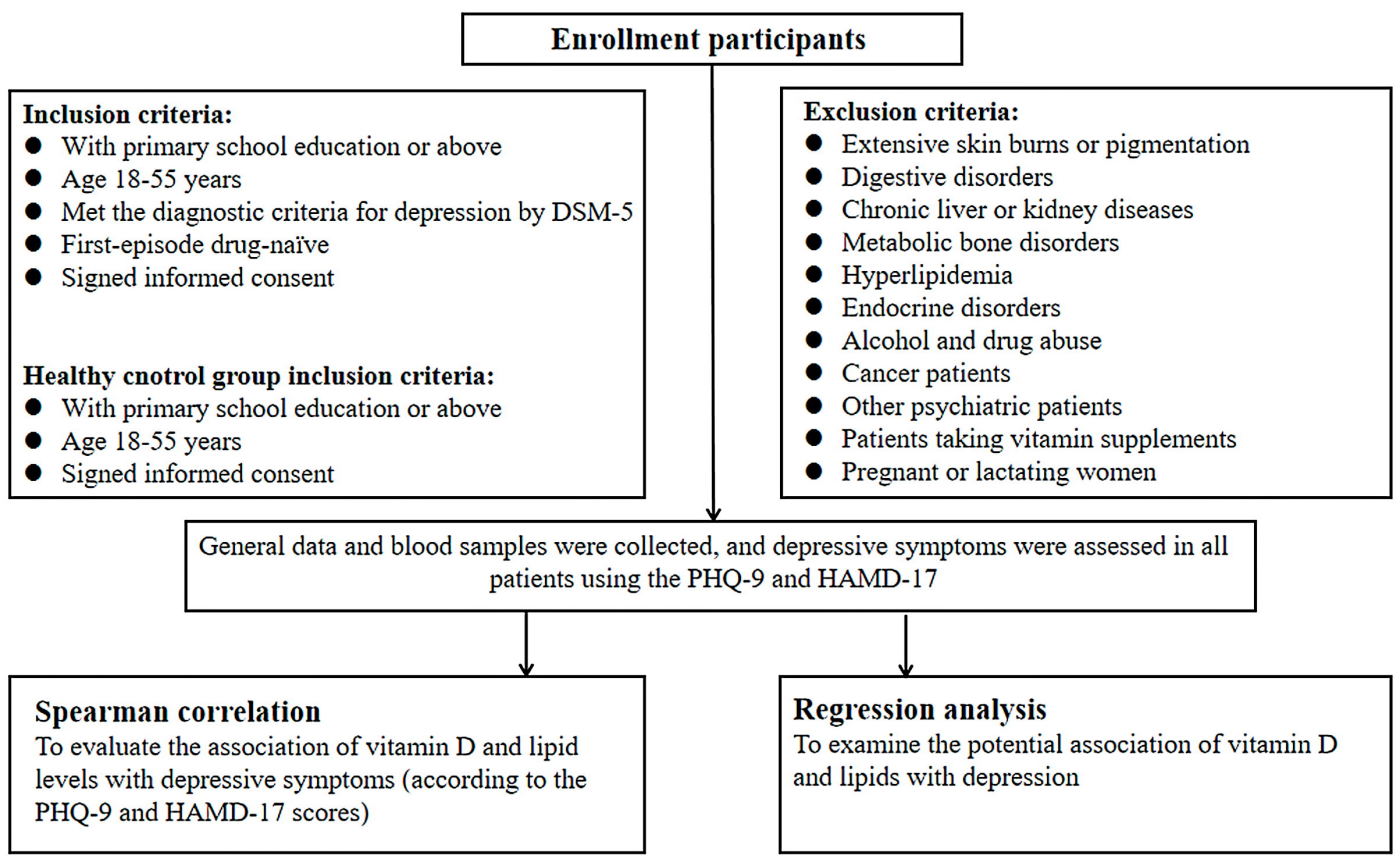
**Study flowchart**. DSM-5, Diagnostic and Statistical Manual of Mental Disorders; PHQ-9, Patient Health Questionnaire-9; HAMD-17, 17-item Hamilton Depression Rating Scale.

Participants aged 18 to 55 were recruited from Hubei Province, China. This study was approved by the Ethics Committee of Renmin Hospital of Wuhan University (approval no.WDRY2020-K191). All patients were enrolled from the outpatient clinic via convenience sampling and provided written informed consent. Depression was diagnosed by an experienced psychiatrist according to the criteria of the fifth edition of the Diagnostic and Statistical Manual of Mental Disorders (DSM-5). Healthy controls were recruited from individuals attending the hospital’s health examination centre. The inclusion criteria comprised: (1) age between 18 and 55 years, (2) an education level of junior high school or above, and (3) provision of signed informed consent. The exclusion criteria comprised potential confounding factors, including extensive skin burns or pigmentation, digestive disorders, chronic liver or kidney diseases, metabolic bone disorders, endocrine diseases, hyperlipidaemia, alcoholism, drug abuse, cancer, comorbid mental disorders, use of vitamin supplements, and pregnancy or lactation (see Fig. 1 for details). Participants in the two groups were matched prospectively based on three key variables: (i) age (in 10-year strata), (ii) sex, and (iii) Body Mass Index (BMI) category (defined by Chinese standards: underweight, normal, overweight, obese). Accordingly, during recruitment, healthy controls were enrolled in a manner that dynamically mirrored the distribution of already-enrolled patients across these matching strata. The aim was to achieve comparable overall group-level distributions of these characteristics, rather than pairwise individual matching. To formally verify group equivalence, a post-hoc matching analysis was performed using calipers for age (±3 years) and BMI (±1 kg/m^2^), along with exact matching on sex. This process did not exclude any already enrolled participants to improve balance.

### 2.2 Measures

A standardised clinical information collection form was used to record patient data, including sex, age, residence, and BMI. Depressive symptoms were assessed using the PHQ-9 and HAMD-17. The PHQ-9 is a self-rated depression scale, with each item scored from 0 to 3 based on the patient’s condition over the past two weeks (total score range: 0–27). The HAMD-17 is an observer-rated scale completed by two medical students trained for inter-rater consistency. It consists of 17 items, with a total score range of 0–53. Higher scores on both scales indicate more severe depressive symptoms. Both the HAMD-17 and PHQ-9 demonstrated good internal consistency in our sample, with Cronbach’s alpha coefficients of 0.915 and 0.940, respectively. An Exploratory Factor Analysis (EFA) was conducted for the HAMD-17. The Kaiser-Meyer-Olkin (KMO) measure was 0.953 and Bartlett’s test of sphericity was significant (χ^2^ = 4066.204, *p* < 0.001), supporting the factorability of the data. For the PHQ-9, the KMO measure was 0.943 and Bartlett’s test of sphericity was significant (χ^2^ = 4266.553, *p* < 0.001), supporting the factorability of the data. Fasting blood samples (5 mL) were collected in tubes containing inert separation gel and coagulant. The samples were left to clot for 30 minutes and subsequently centrifuged at 2000 ×g for 15 minutes. The supernatant serum was then sent to the hospital laboratory for analysis of vitamin D and lipid profiles using liquid chromatography-tandem mass spectrometry (LC-MS/MS). The LC-MS/MS platform consisted of an Ekspert ultraLC 100-XL system and an AB SCIEX 4500 QTRAP mass spectrometer (Applied Biosystems, Foster City, CA, USA). All scale assessments were conducted on the same day as blood collection.

### 2.3 Statistical Analysis

To characterize the study participants, descriptive statistics were presented for all variables. Categorical variables (e.g., sex, ethnicity, residence) were compared between groups using the chi-square test, whereas quantitative variables (e.g., age, BMI, serum vitamin D levels, and lipid profiles) were analysed using the Kruskal-Wallis test due to their non-normal distribution, as confirmed by the Shapiro-Wilk test. Non-parametric tests were employed to assess between-group differences by sex, with statistical significance set at *p* < 0.05. Spearman correlation analysis was used for a preliminary exploration of bivariate relationships among continuous variables, including serum vitamin D, HDL-C, LDL-C, TC, TG, and depression scores (PHQ-9 and HAMD-17) in the depressive group. Binary logistic regression was conducted to identify factors associated with depression, using diagnostic grouping as the dependent variable. Covariates included sex, age, and BMI (potential confounders of lipid metabolism). In the logistic regression analysis, we first performed univariate analyses on sex, age, BMI, vitamin D, TG, TC, LDL-C, and HDL-C. Only vitamin D and HDL-C showed statistical significance (*p* < 0.05). Subsequently, these two variables were included in the multivariate logistic regression model. We performed all statistical analyses with SPSS for Windows (version 26.0, IBM Corp., Armonk, NY, USA), and data visualisation was generated with GraphPad Prism (version 9.0, GraphPad Software, San Diego, CA, USA).

## 3. Results

**Fig. 2.**
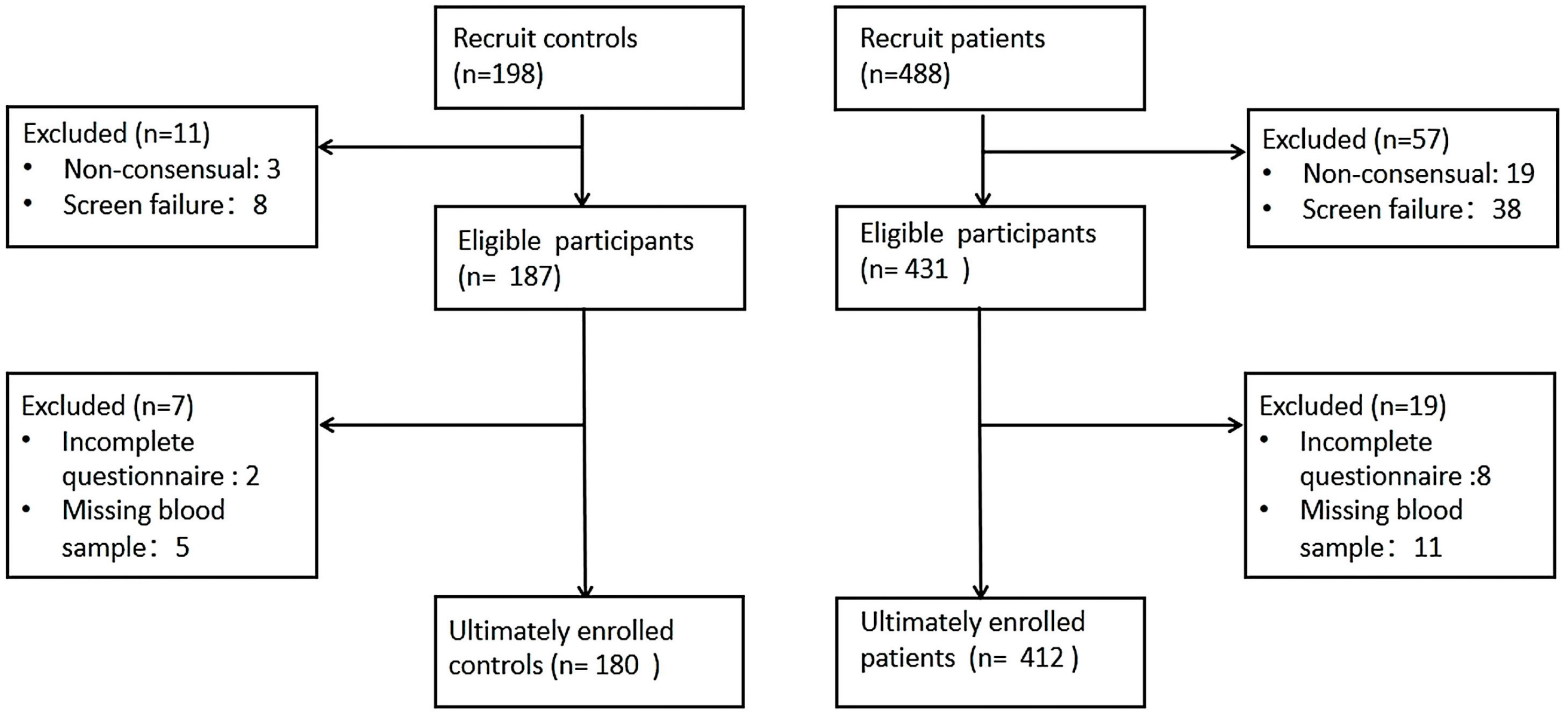
**Participants recruitment flowchart**.

Of the 686 participants initially recruited (198 healthy controls and 488 drug-naïve patients with first-episode depression), 94 were excluded for the following reasons: 22 could not provide informed consent, 46 did not meet the inclusion criteria, and 26 failed to complete all scale assessments and blood draws. Consequently, a final cohort of 180 healthy controls and 412 depressed patients was included in the study (see Fig. 2 for details).

Demographic characteristics for both groups are detailed in Table 1.

**Table 1.** **Participants’ characteristics at baseline (n = 592)**.

Characteristic		Healthy	Depression	*p*
N		180	412	
Sex, n (%)				0.479
	Male	45 (25.0%)	92 (22.3%)	
	Female	135 (75.0%)	320 (77.7%)	
Ethnicity, n (%)				0.130
	Han	165 (91.7%)	391 (94.9%)	
	Ethnic minority	15 (8.3%)	21 (5.1%)	
Residence, n (%)				0.376
	Urban	137 (76.1%)	327 (79.4%)	
	Rural	43 (23.9%)	85 (20.6%)	
Age		22 (21, 24)	21 (20, 25)	0.575
BMI		20.32 (19.15, 22.08)	20.31 (18.73, 22.33)	0.349
PHQ-9		1 (0, 3)	17 (14, 21)	< **0.001**
HAMD-17		1 (0, 2)	20 (16, 24)	< **0.001**
HDL-C (mmol/L)		1.46 (1.24, 1.70)	1.36 (1.18, 1.56)	**0.001**
LDL-C (mmol/L)		2.35 (2.04, 2.80)	2.37 (2.01, 2.74)	0.761
TC (mmol/L)		4.32 (3.89, 4.76)	4.21 (3.78, 4.72)	0.129
TG (mmol/L)		1 (0.71, 1.36)	0.92 (0.71, 1.29)	0.276
Vitamin D (ng/mL)		13 (9, 18)	12 (9, 16)	**0.021**

Data are presented as median (P50) and interquartile range (P25, P75). Bold values indicate statistical significance. BMI, Body Mass Index; HDL-C, high-density lipoprotein cholesterol; LDL-C, low-density lipoprotein cholesterol; TC, total cholesterol; TG, triglyceride.

The two groups did not differ significantly regarding sex, ethnicity, residence, age, or BMI. The overall sample medians were as follows: age, 22 years; BMI, 20.31 kg/m^2^. Compared to the depression group, the healthy control group exhibited significantly lower PHQ-9 and HAMD-17 scores (*p* < 0.001), and notably higher HDL-C and vitamin D levels (*p* = 0.001 and *p* = 0.021, respectively). However, no significant between-group differences were detected in LDL-C, TC, or TG levels (*p* > 0.05).

**Fig. 3.**
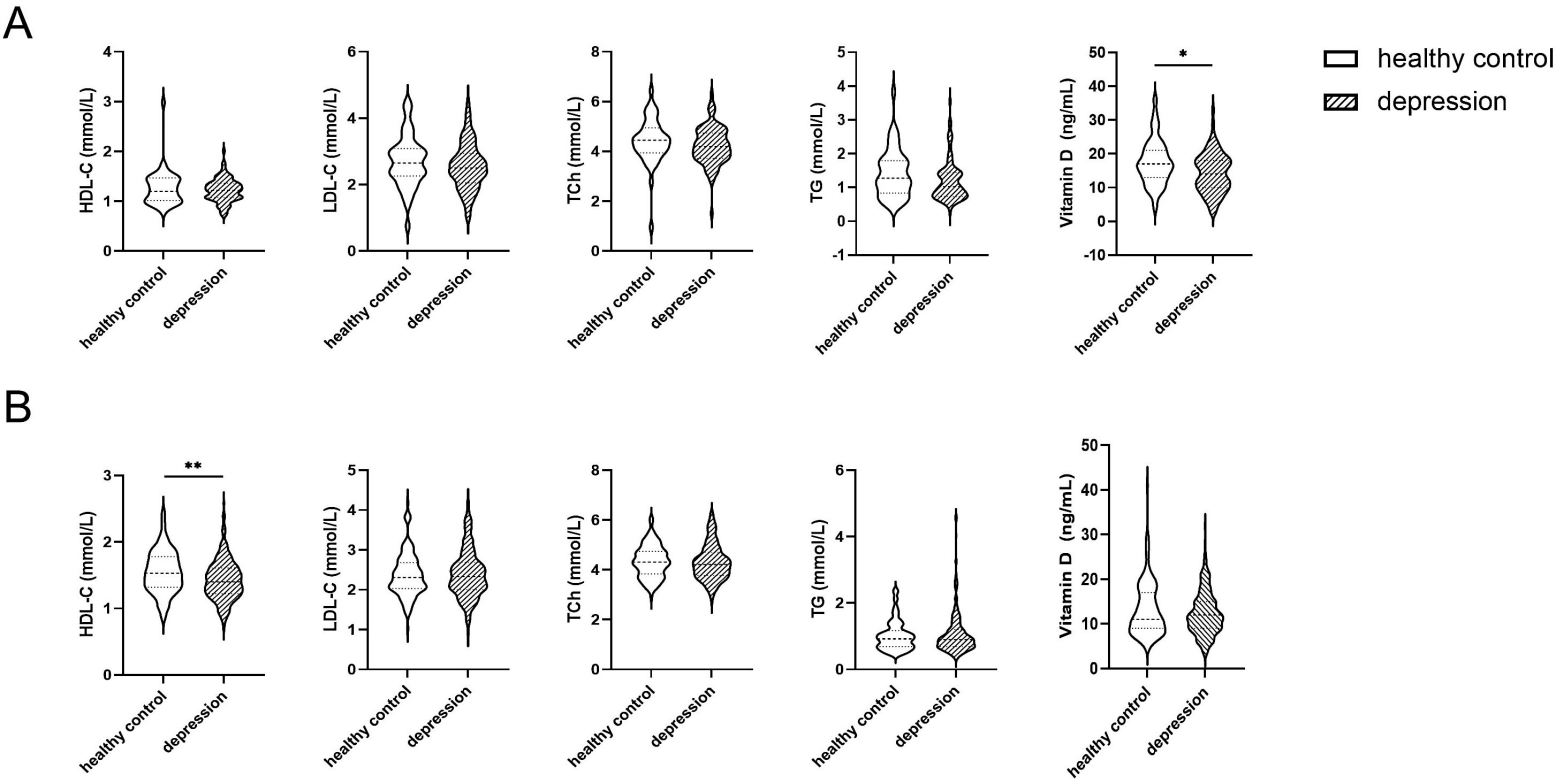
**Violin plots of intergroup differences in vitamin D and lipid profiles by sex**. (A) illustrates the differences in vitamin D and lipid levels between the healthy control group and the depression group in males, whereas (B) presents the corresponding comparisons in females. *p < 0.05, **p < 0.001.

Following stratification by sex, between-group comparisons were conducted. As demonstrated in Fig. 3A, male participants in the depression group exhibited significantly lower vitamin D levels compared to the healthy control group (*p* = 0.013). Fig. 3B reveals that female participants in the depression group showed significantly reduced HDL-C levels relative to the healthy control group (*p* < 0.001). No other significant differences in lipid levels were observed between groups (*p* > 0.05).

As presented in Table 2, Spearman’s correlation analysis demonstrated that vitamin D levels in depressed patients exhibited significant negative correlations with both HAMD-17 scores (r = –0.179, *p* < 0.001) and PHQ-9 scores (r = –0.180, *p* < 0.001). Similarly, TG levels showed inverse relationships with depression severity scores (HAMD-17: r = –0.141, *p* = 0.004; PHQ-9: r = –0.120, *p* = 0.015). Conversely, no statistically significant associations were found between depression scores and HDL-C, LDL-C, or TC levels (*p* > 0.05).

**Table 2.** **Associations between vitamin D, lipid levels, and HAMD-17/PHQ-9 scores in patients with depression (n = 412)**.

vs.	HAMD-17		PHQ-9	
	r	*p* value (2-tailed)	r	*p* value (2-tailed)
HDL-C	0.068	0.167	–0.016	0.739
LDL-C	–0.029	0.562	–0.034	0.491
TC	–0.027	0.578	–0.043	0.388
TG	–0.141	**0.004**	–0.120	**0.015**
Vitamin D	–0.179	< **0.001**	–0.180	< **0.001**

Spearman’s correlation analysis was performed, with the coefficient (r) and statistically significant values shown in bold.

As presented in Table 3, following adjustment for sex, age, and BMI, the analysis revealed two significant protective associations: participants with elevated HDL-C levels exhibited a substantially reduced likelihood of depression compared to those with lower levels (adjusted OR = 0.317, 95% CI: 0.173–0.583, *p* < 0.001). Higher vitamin D levels were similarly associated with decreased depression likelihood (adjusted OR = 0.950, 95% CI: 0.920–0.982, *p* = 0.002). These findings carry the implication that both HDL-C and vitamin D may serve as factors inversely associated with depression.

**Table 3.** **Factors associated with depression in logistic regression analysis (n = 592)**.

Variables	OR (95% CI)	*p*
HDL-C	**0.317 (0.173–0.583)**	< **0.001**
LDL-C	0.948 (0.709–1.266)	0.717
TC	0.865 (0.674–1.109)	0.251
TG	0.917 (0.675–1.246)	0.579
Vitamin D	**0.950 (0.920–0.982)**	**0.002**

Bold values indicate statistical significance. Logistic regression analyses were performed for LDL-C, TC, and TG, adjusted for sex, age, and BMI; for Vitamin D and HDL-C (which showed initial *p* < 0.05), a subsequent analysis including both variables was conducted, also adjusted for sex, age, and BMI.

## 4. Discussion

Our study comprised 412 patients with first-episode, drug-naïve depression and 180 healthy controls, with rigorous exclusion criteria for physical comorbidities, recurrent episodes, and any history of medication or treatments. The analysis revealed significantly higher levels of both HDL-C (which facilitates reverse cholesterol transport and clearance) and vitamin D in healthy controls compared to depressed patients. When stratified by sex, male patients in the depression group exhibited significantly lower vitamin D levels than healthy male controls. In contrast, female patients showed significantly reduced HDL-C levels compared to healthy female controls. Notably, vitamin D levels in females were consistently lower than in males. This disparity may be attributed to women’s greater use of cosmetics or sun protection measures, which can limit sunlight exposure, as well as their relatively lower engagement in outdoor activities compared to males.

Sex, age, and BMI, known determinants of lipid metabolism [24, 25], were included as covariates in our modelling analysis. The results suggested that HDL-C and vitamin D may confer a reduced risk of depression, implying their potential role as protective factors against the disorder. This observation is consistent with prior studies demonstrating that elevated lipid levels, particularly TC and LDL-C (which facilitate cholesterol delivery to peripheral tissues), correlate with more severe depressive symptoms [17, 18]. Conversely, a specific correlation exists between decreased HDL-C levels and symptoms of depression [26, 27] and may function as a predictive biomarker for depression severity [28]. Similarly, in patients with obsessive-compulsive disorder (OCD), lower HDL-C levels have been linked to increased suicidal ideation [29].

A bidirectional relationship exists between vitamin D and lipids. Firstly, as a fat-soluble vitamin, vitamin D relies on dietary lipids for dissolution and intestinal absorption. Secondly, through binding to its receptor and modulating gene expression [11, 12], vitamin D influences cholesterol homeostasis and fatty acid metabolism, thereby regulating blood lipid concentrations. Epidemiological studies have demonstrated that individuals with vitamin D deficiency frequently present with elevated TC and LDL levels, with this deficiency being particularly prevalent in obese populations [30]. Furthermore, vitamin D shows an inverse association with circulating lipid levels, and supplementation has been shown to exert beneficial effects on lipid profiles [31]. Interestingly, weight reduction has also been associated with improved vitamin D status [32]. In summary, the vitamin D-lipid interaction is reciprocal: while vitamin D requires lipids for absorption and storage, it simultaneously plays a crucial role in lipid metabolism. However, confounding factors must be considered. For example, decreased physical activity and reduced sunlight exposure, both common among patients with depression, may independently contribute to lower vitamin D levels alongside elevated TC and TG concentrations.

The roles of vitamin D and lipids in depression, however, involve greater complexity. Our study identified an inverse association between vitamin D levels and depressive symptoms, consistent with previous research findings [5]. The immune-inflammation hypothesis, linking dysregulated inflammation to neural circuit and neurotransmitter alterations in depression, has gained substantial support [33]. Notably, depression, dyslipidaemia, cardiovascular disease, and insulin resistance share common immune-inflammatory alteration [33, 34]. Vitamin D regulates inflammatory cytokine production and suppresses pro-inflammatory cell proliferation [35]. Through vitamin D receptor (VDR) signalling, it suppresses NLRP3-mediated immune responses [36]. Chronic inflammation is often associated with elevated cholesterol and triglyceride levels [37]. Studies indicate that in human monocytes, lipids activate the NLRP3 inflammasome. This activation promotes caspase-8 maturation through a cascade involving Lyn/Syk-dependent calcium influx and the generation of reactive oxygen species.

In addition to its anti-inflammatory effects, vitamin D modulates brain neuroplasticity, neurotransmitter biosynthesis, neuroprotection, and synaptic transmission by regulating neurotrophic factors and redox signalling pathways [38, 39, 40]. A study in pregnant rats demonstrated that vitamin D deficiency led to offspring with reduced cortical thickness and enlarged lateral ventricles [41, 42]. Furthermore, vitamin D deficiency alters neuronal morphology, impairing neurite outgrowth, branching, and periaqueductal length, which may contribute to speech and cognitive dysfunction [43, 44]. Research has also shown that a high-fat diet (HFD) disrupts hippocampal synaptic plasticity, reducing dendritic spine density and impairing long-term potentiation (LTP) [45, 46]. Chronic HFD exposure in adult mice leads to a reduction in key hippocampal neurotrophic factors, such as brain-derived neurotrophic factor (BDNF) [47, 48, 49]. Notably, these neurobiological alterations are frequently observed in depression [50, 51, 52, 53].

In summary, lipids are not only essential components of cell membranes but also play a pivotal role in nervous system function. Dysregulated lipid metabolism has been linked to neurodegenerative and psychiatric disorders, whereas vitamin D may indirectly support neural health and plasticity through its modulation of lipid metabolism. Therefore, maintaining an equilibrium between these factors is critical for optimal neurological function.

In our study, discrepancies were observed between correlation and regression analyses. Several factors may explain this inconsistency: First, correlation analysis exclusively measures linear relationships; when variables exhibit nonlinear associations (e.g., curvilinear patterns), the correlation coefficient may approach zero. Second, outliers may disproportionately affect correlation results, whereas regression analysis typically reduces their influence through least squares estimation or robust methods. Additionally, while bivariate correlation examines pairwise relationships, regression analysis simultaneously evaluates the effects of multiple independent variables on the dependent variable. Importantly, some variables may demonstrate modified influence patterns after covariate adjustment. In our study, regression models controlled for sex, age, and BMI, key covariates known to influence lipid metabolism, thereby strengthening the validity of our results.

### Limitations

These results should be interpreted in light of certain study limitations. First, the cross-sectional design precludes causal inference. The most critical limitation of this study is the lack of measurement and adjustment for several important confounding variables, such as smoking, dietary habits, and physical activity. These factors are strongly associated with vitamin D levels, lipid profiles, and the risk of depression. Due to the presence of these unmeasured confounders, the observed associations in this study may be subject to unpredictable bias (potentially overestimated or underestimated), which severely limits the internal validity of our findings and precludes any causal inference. For example, physical inactivity could concurrently lead to lower vitamin D levels (due to reduced outdoor sun exposure), decreased HDL-C levels, and an increased depression likelihood, leading to an overestimation of any observed inverse associations. Conversely, a diet rich in fatty fish may simultaneously raise both vitamin D and HDL-C levels while also being associated with a lower depression likelihood, which could introduce a similar inflationary bias. However, the possibility of negative confounding also exists. For instance, obesity—as an inflammatory state—is often associated with lower vitamin D levels and a higher depression risk, yet its relationship with conventional lipid profiles is complex; if not adequately adjusted for, it might partially mask the true associations. Given these competing directions of bias, the direction and magnitude of the net association we observed remain uncertain. Future studies must prioritize the collection of these key variables to provide more reliable estimates. Finally, it should be acknowledged that seasonal variations in vitamin D levels were not accounted for in this study, despite their potential impact on the findings. Future longitudinal studies incorporating repeated measures would help clarify the temporal relationships between vitamin D, lipid metabolism, and depression onset and progression. Our ultimate objective remains the identification of reliable biological markers for depression.

## 5. Conclusions

Serum vitamin D and HDL-C levels were significantly lower in patients with depression compared to healthy controls. Moreover, a negative correlation was observed between serum vitamin D concentrations and the severity of depressive symptoms, indicating that lower vitamin D levels were associated with more severe clinical manifestations of depression. These findings collectively suggest that both vitamin D and HDL-C may be inversely associated with depression, likely involved in the underlying biological pathways related to depression.

## Availability of Data and Materials

The data that support the findings of this study are available from the corresponding authors upon reasonable request.
